# Recent Progress of Dopamine-Modified Tissue Adhesives for Biomedical Applications in Underwater Environments

**DOI:** 10.1007/s13770-026-00804-6

**Published:** 2026-03-19

**Authors:** Chong-Su Cho, Gi-Yeon Han, Eun Byul Koh, Yo-Han Kim, Yeon Ho Je, Hyun-Joong Kim

**Affiliations:** 1 Department of Agricultural Biotechnology, Research Institute of Agriculture and Life Sciences, Seoul National University, Seoul, 08826 Republic of Korea; 2 Department of Chemical Engineering, Chung-Ang University, Seoul, 06974 Republic of Korea; 3 Program in Environmental Materials Science, Department of Agriculture, Forestry and Bioresources, Seoul National University, Seoul, 08826 Republic of Korea

**Keywords:** Tissue adhesives, Biomedical application, Underwater environments

## Abstract

**Background:**

Underwater adhesion of polymeric adhesives is highly desirable in specific applications such as wound dressings, wearable devices, bioelectronic devices, biosensors, and water pipeline leakage repairing. However, underwater bonding is considerably different from bonding in air because interfacial water molecules substantially weaken the intimate contact adhesion between the adhesive and submerged surfaces, thus significantly limiting the application of adhesives in various fields.

**Method:**

This review was compiled by searching relevant references on PubMed database (before April 2025) based on selected keywords.

**Results:**

Recently, many wet adhesion technologies and diverse and flexible adhesive materials have been employed to address the weak adhesion strengths and inferior mechanical properties in underwater environments. Among several strategies, mussel-inspired catechol-based underwater adhesion has gained the attention of scientists because mussel-inspired tissue adhesives (TAs) demonstrate numerous advantages including many interactions with substrates, various designs of some interesting smart TAs, and excellent adhesion based on several interfacial interactions dominated by 3,4-dihydroxyphenylalanine, a catecholic amino acid in mussel adhesive proteins.

**Conclusion:**

We discuss the mechanism of catechol-based underwater adhesion, classification of underwater adhesives, and characteristics, applications, advantages, and disadvantages of dopamine (DA)-modified polymeric TAs. Furthermore, we review stimuli-responsive TAs and the essential factors affecting the adhesions of DA-modified TAs in underwater environments. Finally, we discuss some current technical challenges and future perspectives for underwater adhesion.

## Introduction

Recently, the use of tissue adhesives (TAs) has attracted the attentions of scientists and industrialists due to several advantages, including blood leakage prevention, shorter surgery time, less pain, infection mitigation, and no requirement for removal procedures, of TAs [1]. Traditional and invasive techniques, such as sutures, staples, clips, and skin closure strips, have been used for wound closure because they stop bleeding, prevent blood leakage, and restore tissue structure and function [2]. Underwater adhesion of TAs is highly desirable in specific applications, for example, biomaterials, such as wound dressings [3, 4], antibacterial agents [5], wearable devices [6], bioelectronic devices [7], biosensors [8], and water pipeline leakage repairing [9]. However, underwater bonding is considerably different from bonding in air because interfacial water molecules substantially weaken the intimate contact adhesion between the adhesive and submerged surfaces, thus significantly limiting the application of adhesives in various fields [10]. Recently, many wet adhesion technologies and diverse and flexible adhesive materials have been used to address the weak adhesion strengths and inferior mechanical properties of adhesives in underwater environments. Several strategies have been reported for realizing underwater adhesion, and breakage of the hydration layer and interaction of the adhesive with the substrate surface designed at different scales is one of the main approaches to achieve wet adhesion [11]; organic solvents, monomers, and polymers can be utilized to remove the hydration layer and realize surface wetting to achieve dehydration at the nanometer level [12]; water-absorbing fillers, including inorganic materials and hydrophilic polymers, can be used for wet surfaces [13]; surface microstructures promote water drainage, thereby inhibiting permanent water trapping at large length scales [14], via interfacial bonding caused by the formation of covalent or non-covalent bonds between the surface microstructures and substrate surface after dehydration; moreover, interfacial bonding can be realized via covalent bonds for substrates with reactive functional groups and non-covalent bonds for substrates without reactive functional groups [15]. Compared to conventional TAs, mussels, which are marine organisms, can adhere to various substrate surfaces using a proteinaceous holdfast to produce a strong bond with the substrates; mussel-inspired TAs demonstrate several advantages such as many interactions with the substrates, various designs of some interesting smart TAs, and excellent adhesion based on numerous interfacial interactions dominated by 3,4-dihydroxyphenylalanine (DOPA), a catecholic amino acid in mussel adhesive proteins [10]. Herein, we discuss the mechanism of catechol-based underwater adhesion, classification of underwater adhesives, and characteristics, applications, and advantages, and disadvantages of dopamine (DA)-modified polymeric TAs. Additionally, we review stimuli-responsive TAs and the essential factors affecting the adhesions of DA-modified TAs in underwater environments. Finally, we discuss some current technical challenges and future perspectives for underwater adhesion.

## Mussel-inspired catechol-based underwater adhesion

### Mechanism of catechol-based underwater adhesion

**Fig. 1:**
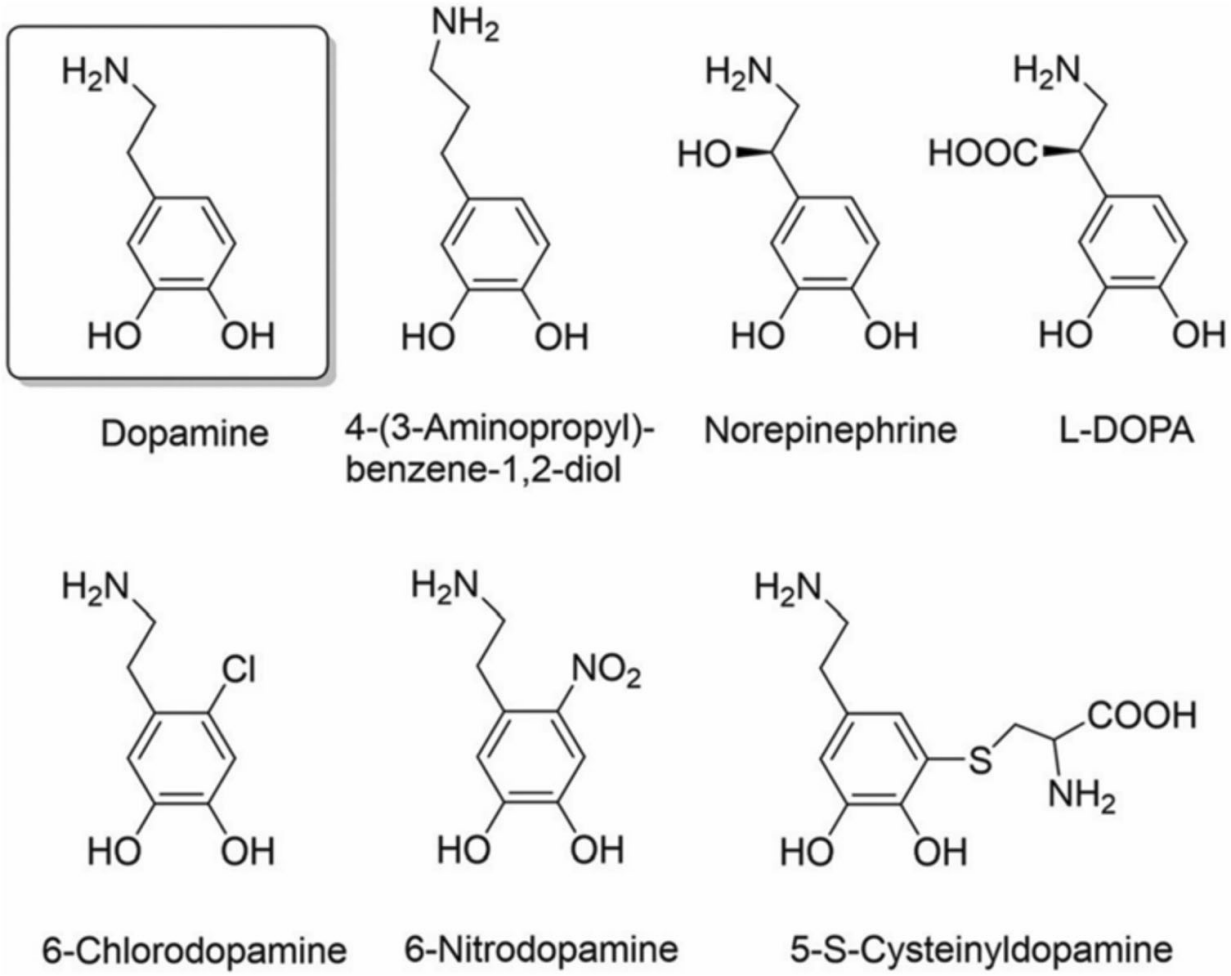
Chemical structure of DA and common DA derivatives. [Adapted from Barros NR et al. (2021) with permission from the Royal Society of Chemistry]

**Fig. 2:**
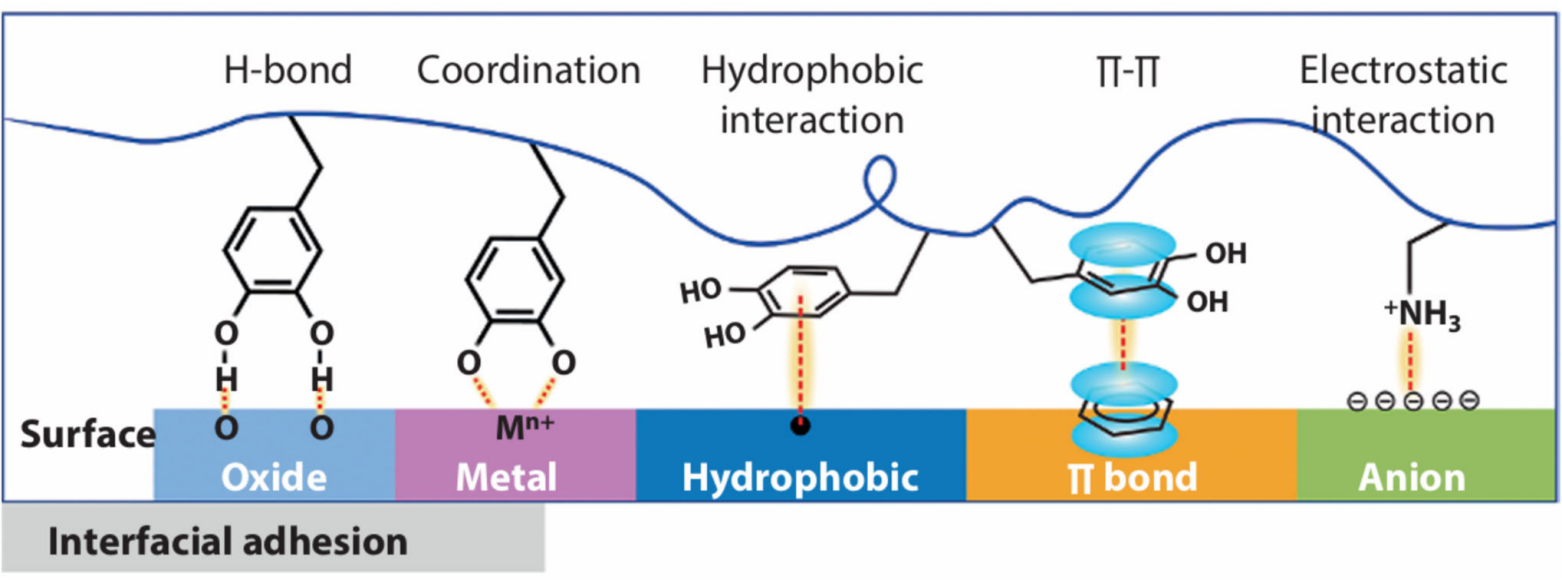
Adhesion mechanisms of the mussel foot proteins interfacial adhesion. [Adapted from Yanfei M et al. (2021) with permission from John Wiley and Sons.]

**Fig. 3:**
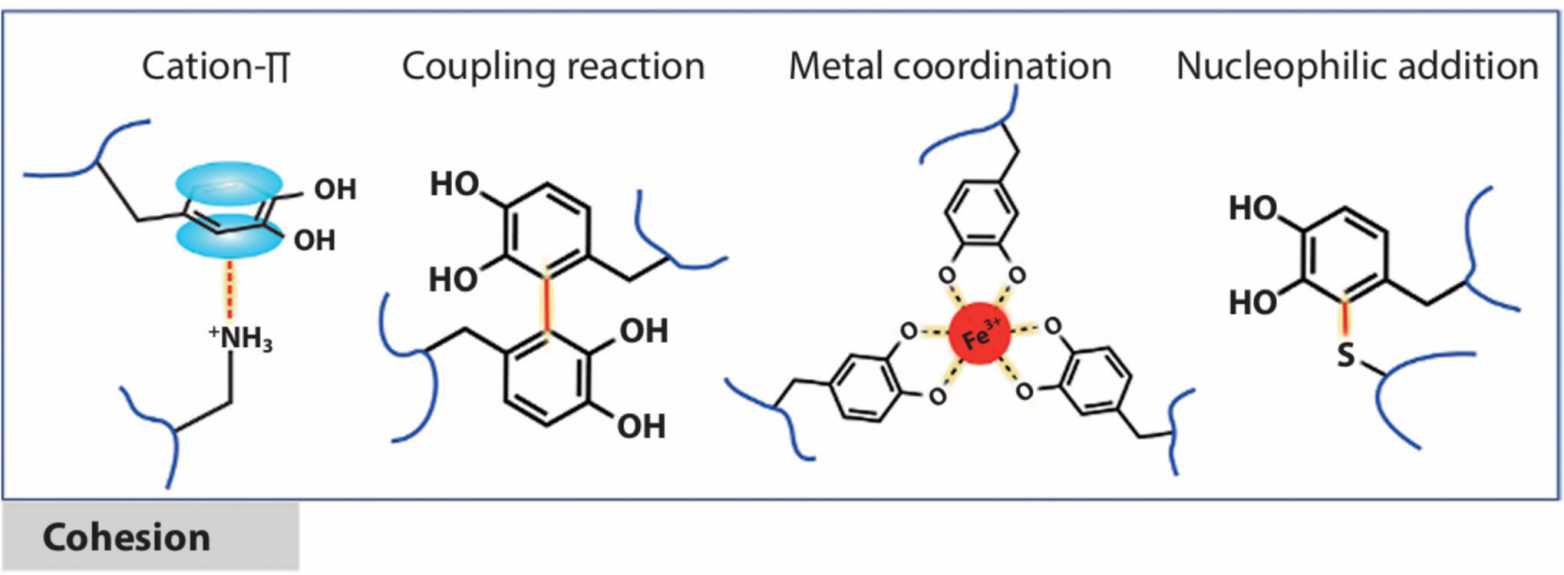
Adhesion mechanisms of the mussel foot proteins cohesion. [Adapted from Yanfei M et al. (2021) with permission from John Wiley and Sons.]

Ability of mussels to adhere to various surfaces under wet conditions has attracted considerable attention because these abilities originate from the catecholic amino acid of DOPA secreted by the feet of mussels [16]. Catechol groups in polydopamine (PDA) play critical roles in adhesion because of the penetrating water boundary layers. Nevertheless, PDA formation from DA and its derivatives, as shown in Fig. 1, [17] still needs to be elucidated [18]. Catechol groups are reduced and converted to O-quinone groups under alkaline conditions, followed by reaction with the thiol and amino groups of the substrates [19]. Furthermore, they react with metal ions, including Fe^3+^, Cu^2+^, and Zn^2+^, contributing to PDA adhesion [20]. Consequently, the catechol moieties of DOPA have been investigated as an important inspiration for the design of underwater adhesives [21]. Moreover, mussel foot proteins comprise several functional groups, which can exhibit various interactions such as H-bonding, hydrophobic interaction, metal coordination bonding, and cation/anion/π-π interaction [10]. Therefore, mussels simultaneously demonstrate high interfacial adhesion (Fig. 2) and cohesion (Fig. 3) based on the interaction between proteins and between proteins and substrates.

### Classification of current underwater adhesives

**Fig. 4:**
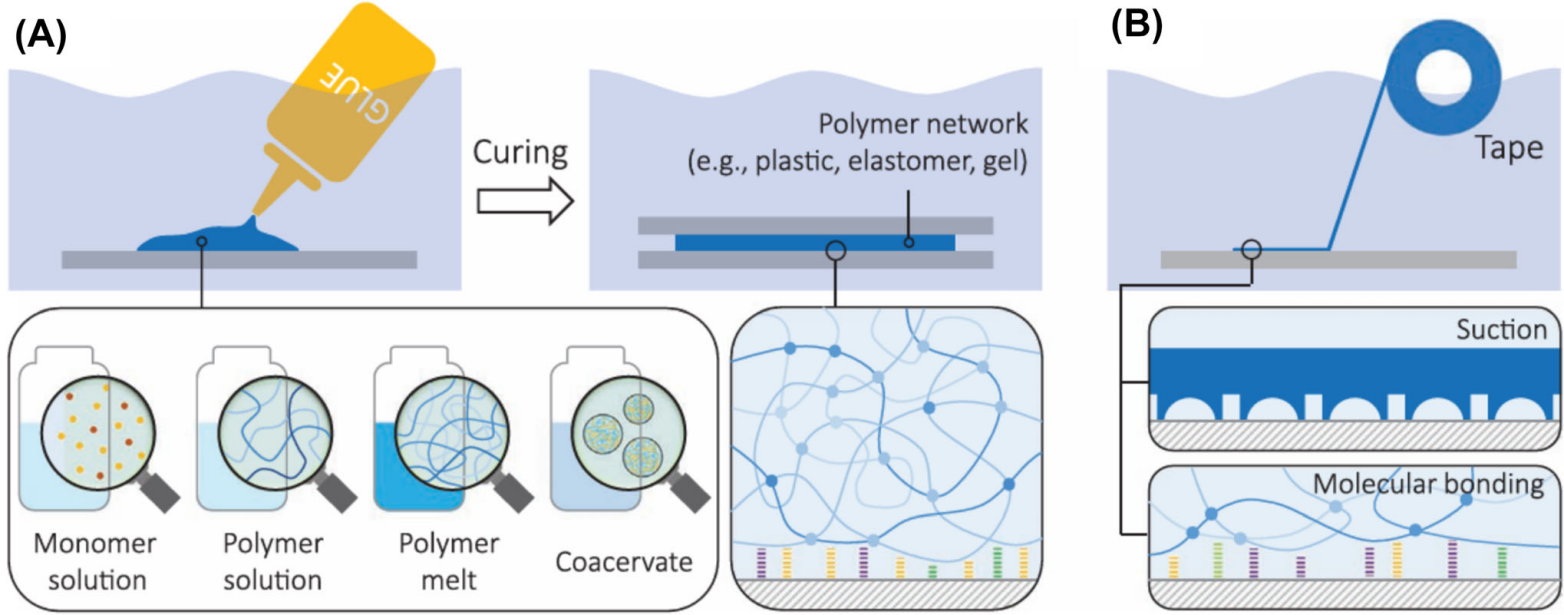
Current underwater adhesives. **A** Glue-type underwater adhesives, which are in liquid form and require a curing process to solidify. The liquid can be monomer solutions, polymer solutions, polymer melts, coacervates, or their mixtures. **B** Tape-type underwater adhesives, which are soft viscoelastic elastomers or gels. Glue-type adhesives form full molecular bonding with the substrate, thus demonstrating strong and irreversible adhesion, whereas tape-type adhesives usually have very weak but reversible adhesion due to the difficulty of forming molecular bonding in water. [Adapted from Fan H et al. (2021) with permission from John Wiley and Sons.]

Generally, underwater adhesives can be classified into two major categories according to the bonding methods: glue-type adhesives (GTAs) and tape-type adhesives (TTAs) (Fig. 4) [22]. GTAs prepared from liquid precursor solutions, including monomer solutions, polymer solutions, polymer melts, and coacervates, are polymerized and/or cross-linked to generate solids via molecular-level interfacial interactions [22]. Typically, the bonding strengths of GTAs are higher than the cohesion strengths; however, the curing times are long and bonding is irreversible. GTAs usually utilize chemical structures for solidification and curing to obtain strong cohesion [22]. In contrast, TTAs, as soft solids, directly adhere to wet surfaces via molecular interactions and/or physical suction; nevertheless, their bonding strengths are weak, and they exhibit instant and reversible adhesion due to inadequate interfacial contact resulting from inefficient dehydration and roughness of the substrate surface. TTAs generally focus on dehydration and formation of strong interfacial bridging; however, they involve different length scales [22].

### DA-modified biomaterials and their applications

Typically, DA-modified biomaterials can be prepared by conjugating DA via its active groups, such as amino and phenolic hydroxyl groups, with biomaterials using *N*-(3-dimethylaminopropyl)-*N”*-ethylcarbodiimide hydrochloride and *N*-hydroxysuccinimide as coupling agents. In this section, we discuss several biomaterials conjugated with the catechol groups of DA.

#### DA-modified poly(ethylene glycol) (PEG)-based TAs

##### Characteristics of PEG

PEG has been widely used as biomaterials in drug delivery systems and tissue engineering because of its numerous advantages, for instance, hydrophilicity, biocompatibility, easy chemical modification, and non-immunogenicity [23].

##### Applications

*Wound dressing*

**Fig. 5:**
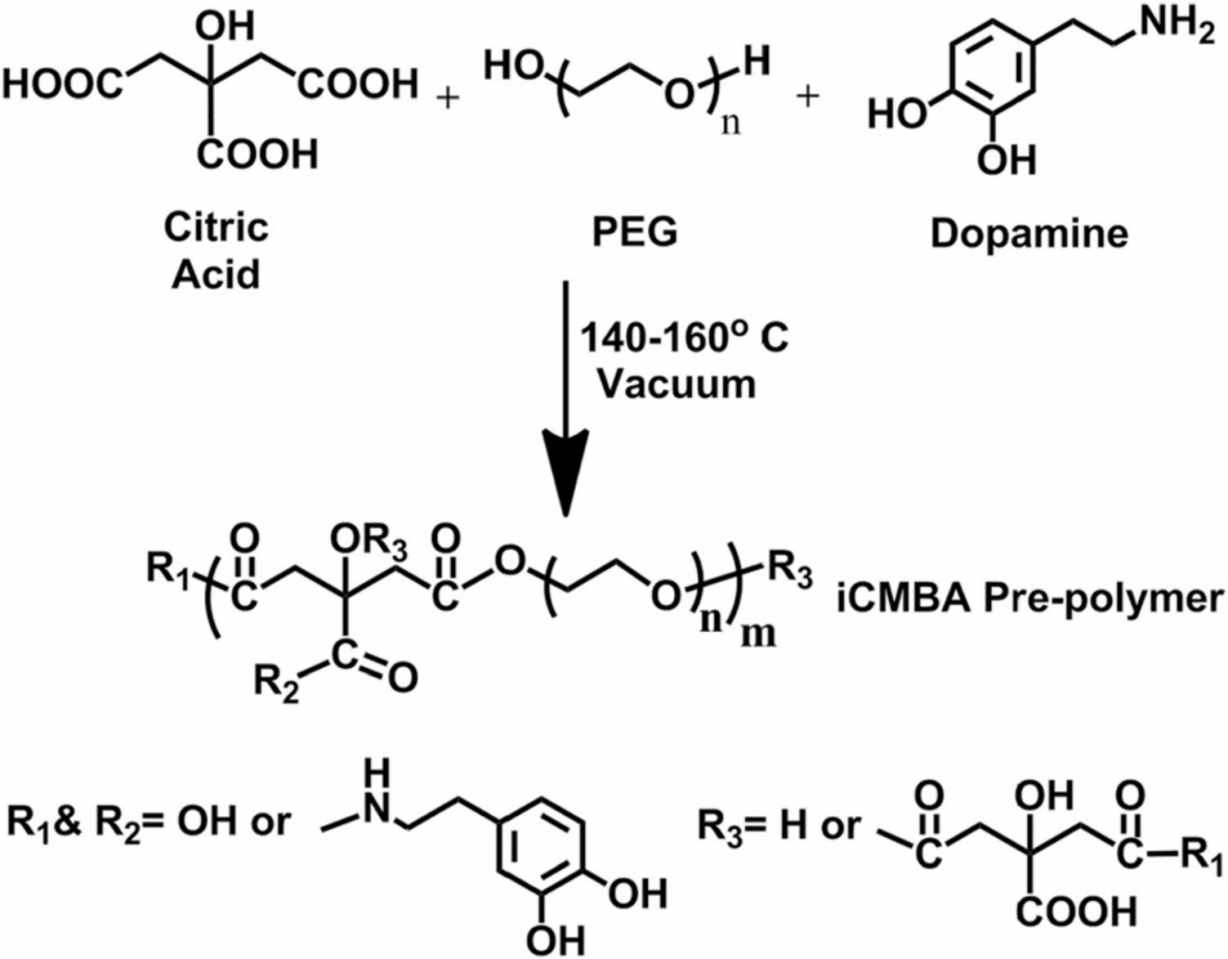
Schematic representation of iCMBA pre-polymers synthesis through polycondensation reaction. [Adapted from Mehdizadeh M et al. (2012) with permission from Elsevier]

Mehdizadeh et al. synthesized citrate-based mussel-inspired PEG-based TAs by reacting PEG, DA, and citric acid to fabricate a prepolymer via polycondensation followed by crosslinking of the prepolymer by mixing it with sodium periodate, as depicted in Fig. 5.[24] Citric acid formed polyesters with PEG and provided pendant carboxyl groups to conjugate DA. These bioadhesives demonstrated 2.5–eightfold higher wet adhesion to porcine small intestine than that of fibrin glue. Additionally, these bioadhesives were applied to close wounds produced on the backs of rats, instantly stopping bleeding and exhibiting excellent tissue compatibility. Moreover, the same group prepared magnesium oxide (MgO)-based mussel-inspired PEG-poly(propylene glycol) (PPG)-PEG-based bioadhesives by reacting PEG-PPG-PEG, DA, and citric acid to generate a prepolymer via polycondensation followed by crosslinking of the prepolymer by mixing it with MgO as both a crosslinking agent and composite filler [25]; PEG-PPG-PEG reduced the swelling of bioadhesives owing to its hydrophobic property, and MgO enhanced the adhesion. Bioadhesives cross-linked by MgO in the presence of sodium periodate as an oxidizing agent demonstrated eightfold higher wet adhesion to porcine intestinal submucosa than that of fibrin glue with high mechanical strength. Furthermore, these bioadhesives were applied to close wounds created on rat skin incisions, exhibiting outstanding biocompatibility *in vitro* and *in vivo*.

*Bone tissue engineering*

Yan et al. prepared mussel-inspired conducting copolymers using aniline tetramer intelligent TAs for bone tissue engineering [26]; conductive TA copolymers obtained by the copolymerization of aniline tetramer methacrylamide, DA methacrylamide, and PEG methyl ether methacrylate were expected to demonstrate multifunctional properties such as conductivity arising from the aniline groups, adhesiveness stemming from the DA groups, and biocompatibility afforded by the PEG groups. Adhesion strengths of these copolymers were 1.28 MPa at 6 mol% aniline due to H-bonding, π-π interactions, and polymer long-chain entanglement. Additionally, osteogenic differentiation, alkaline phosphatase activity, Ca deposition, and relative expression levels of osteogenic genes were significantly enhanced by electrical stimulation, indicating that these TAs are promising for orthopedic and dental applications; nevertheless, their *in vivo* application was not examined.

*Electronic devices*

Song et al. fabricated hydrogel strain sensors composed of genipin-cross-linked gelatin (GE) and DA-modified PEG for *in vivo* monitoring of cardiac function [27]; the two hydrogel compositions exhibited hysteresis-free and highly sensitive strain sensing of ionically conductive property, and the resulting sensors demonstrated implantability, biocompatibility, and adhesiveness. These hydrogel sensors exhibited little to no hysteresis with 30–50 × higher gauge factors. Moreover, the implantable electronic devices demonstrated impedance-based strain sensing of cardiac output in a porcine model, indicative of the applicability of the abovementioned hydrogel sensors in various implantable electronics. Characteristics of DA-modified PEG-based TAs are presented in Table 1.

**Table 1:** The characteristics of DA-modified PEG-based TAs

Applications	Components	*In vivo* model	Results	References
Wound dressing	PEG, DA, citric acid	Porcine	2,5–8 folds stronger wet adhesion than fibrin glue	[24]
	PEG-PPG-PEG, DA, citric acid, MgO, periodate	Rats	Fast closing of wounds	[25]
Bone tissue engineering	Aniline tetramer methacrlyamide, DA methacrylamide, PEG methyl ether methacrlylate	NT	Having conductivity due to the aniline groups, osteogenic differentiation	[26]
Electronic device	Genipin, GE, DA, PEG	Porcine	Impedance-based strain sensing of cardiac output	[27]

##### Advantages and disadvantages

PEG-based TAs exhibit advantages, for example, biocompatibility and facile chemical modifications of their functionalities. However, inferior mechanical properties, low cohesive strengths with brittleness, high swelling property owing to their hydrophilic natures, and multiple preparation steps for application are the major disadvantages of PEG-based TAs.

#### DA-modified GE-based TAs

##### Characteristics of GE

GE acquired by thermal treatment of collagen has been applied in biomedical applications, such as tissue engineering, cell culture, and pharmaceutical formulations [28], due to its biodegradability, biocompatibility, low immunogenicity, easy modification by chemical crosslinking with other materials, and low cost.

##### Applications

*Hemostasis*

Han et al. fabricated a GE-based TA hydrogel comprising DA-grafted GE and a mixture of phenylboronic acid (PBA) and graphene oxide (GO) by oxidative cross-linking between catechol groups and PBA for self-healing hemostasis and electrical conductivity [29]; DA in DA-grafted GE improved adhesion, and the addition of GO enhanced the mechanical properties and electrical conductivity of the hydrogel. This hydrogel demonstrated hemostatic property in a rat hepatic hemorrhage model. Furthermore, the electromyography signals of finger movement were monitored by an adhesive electrode hydrogel monitor, implying the potential applications of TA wearable devices.

Hu et al. prepared an injectable and viscoelastic TA hydrogel composed of DA-grafted GE and PBA-grafted hyaluronic acid (PBA-HA) by cross-linking DA-grafted GE and PBA-HA via boronated ester bonds for the treatment of brain lesions [30]; this self-healing hydrogel mimics the composition, stiffness, and viscoelasticity of native brain parenchyma. It exhibited rapid hemostasis, high tissue adhesion, and efficient self-healing in the right cortex of rats when injected into an artificial cavity. Additionally, the hydrogel supported neural cell infiltration, decreased astrogliosis and glial scarring, and closed lesions in a mouse model of brain lesions, suggesting the ability of self-healing hydrogels to tune cellular mechanical microenvironment in brain lesion treatment.

*Wound dressings*

Montazerian et al. synthesized a stretchable GE-based TA hydrogel comprising cross-linked GE methacrylate (GEMA) and PDA for wound dressings and wearable devices [31]; this hydrogel demonstrated outstanding mechanical and adhesive properties, attributed to PDA. Its stretchability and adhesion force in tensile modes on porcine skin were 5.7 and 4 times those of GEMA, respectively, owing to the existence of reactive oxidized quinone species, indicating potential application of this skin-attachable TA hydrogel.

Cheng et al. prepared sprayable TA hydrogels composed of DA-conjugated cross-linked GEMA, cerium oxide (CO) nanoparticles (NPs), and antimicrobial peptide (AMP) for wound dressing [32]; DA-conjugated cross-linked GEMA improved the binding affinity of the hydrogels to the wet skin surface, release of AMP from the hydrogels provided contact ablation against bacteria, and CO NPs exhibited reactive oxygen species (ROS)-scavenging properties. These hydrogels demonstrated sprayability, adhesiveness, antibacterial activity, and ROS-scavenging ability *in vitro*. Moreover, they facilitated the most rapid healing and promoted skin restoration in a rat skin defect model, implying their promising application in wound dressing.

An et al. fabricated a drug-loaded Janus mucosal dressing comprising PDA, GE, and nanoclay by forming a stable network structure between GE and PDA via Michael addition and Schiff base reaction for healing oral ulcers [33]; GE/PDA/nanoclay controlled adhesion and toughness of this dressing via synergistic physical and chemical interactions among GE, DA, and nanoclay. This dressing exhibited a strong wet adhesion force of 53 kPa and high toughness of 10,261 ± 00 J m^−1^ with high cell adhesion and proliferation *in vitro*. Additionally, dressing facilitated the healing of oral ulcers in Sprague–Dawley (SD) rats due to its strong mucosal adhesive property and therapeutic effect of the loaded drug, indicating potential applicability of this mucosal dressing in drug delivery systems.

Liu et al. prepared stretchable and breathable TA nanofibrous hydrogels composed of DA and GEMA by *in situ* hybrid photo-cross-linking of electrospun nanofibers for wound dressing [34]; the balance of adhesion and cohesion based on photo-cross-linking of methacrylate anhydride (MA) groups in GEMA and physical/chemical reaction between DA and GEMA are very important. These hydrogels demonstrated 2 times higher tensile strengths and 2.3 times higher adhesive strengths on porcine skin than those of the GE nanofibrous hydrogels. Furthermore, they accelerated healing of skin defects on the backs of mice and exhibited stretchability and breathability, implying their promising applicability in wound dressings.

Li et al. synthesized self-adhesive sponges comprising DA-grafted GEMA, quaternized chitosan (QCS), and glycerin for wound dressing [35]; DA-grafted GEMA offered excellent self-adhesive ability to the sponges, QCS endowed the sponges with outstanding blood coagulation and antibacterial activities, and glycerin was used as a plasticizer for rendering the sponges more flexible. These sponges demonstrated better antibacterial activity than that of a commercial GE hemostatic sponge. Moreover, hemostasis times of the sponges were 33.3 ± 6.7 s, which are better than that of the commercial GE hemostatic sponge, indicating outstanding potentials of these sponges for hemostatic wound dressings.

Wang et al. prepared a mesenchymal stem cell (MSC)-derived extracellular vesicle (EV)-loaded TA hydrogel composed of DA-grafted GEMA and EV by *in situ* photo-cross-linking for diabetic wound healing [36]; DA-grafted GEMA provided tissue adhesiveness to this hydrogel under wet conditions, and MSC-EVs afforded high retention rates at wound sites. This hydrogel exhibited high adhesiveness and biocompatibility and promoted cell migration and angiogenesis *in vitro*. Additionally, it demonstrated prominent wound-healing ability with collagen deposition, skin appendage regeneration, and interleukin-6 expression in a skin wound model of diabetic rats, offering a potential approach for the treatment of diabetic wounds.

Lin et al. fabricated durable TA hydrogels comprising GE, silica, and DA by introducing DA into a GE-silica hybrid dressing created via a sol–gel method using 3-glycidoxypropyltrimethoxysilane as a coupling agent for wound dressing [37]; DA boosted adhesiveness, and coupling established covalent bonds between GE and silica, thereby enhancing structural stability. These hydrogels exhibited approximately 2.5 times higher adhesion to porcine skin under wet conditions than that of GE. Furthermore, they significantly increased wound healing rates on the back skins of SD rats, indicating their promise as moist wound dressings.

*Bone defect repair*

Sun et al. prepared a three-dimensional (3D) bioprinted bone marrow-derived MSC (BMSC)-laden TA composed of DA-grafted GE, GEMA, methacrylated silk fibroin (SF), and GO nanosheet for artificial periosteum [38]; GEMA provided excellent adhesive property, and the BMSC-laden SF/GO nanosheet promoted bone defect repair in the 3D bioprinted knitted system [38]. Developed bioink demonstrated adequate printability and satisfactory cell viability. Additionally, 3D bioprinted artificial periosteum constructed using the calvarial areas of SD rats effectively enhanced osteogenesis, affording an outstanding strategy for bone defect repair.

Ma et al. prepared an osteoconductive TA hydrogel comprising DA-grafted GE and PDA-conjugated hydroxyapatite (HY) NPs for bone regeneration [39]; DA-grafted GE offered very high adhesive property under wet conditions, and PDA-coated HY NPs created a highly biomimetic native bone tissue microenvironment owing to their high compatibility. The hydrogel exhibited very high compressive strength without any effect on the microstructure due to high crosslinking density between DA-grafted GE and PDA-coated HY NPs. Moreover, the hydrogel accelerated bone repair efficiency in a rat model of femoral defects, suggesting that it is a potential bone repair biomaterial.

Moazami et al. synthesized multifunctional TA hydrogels composed of PDA, bredigite NPs, and Fe^3+^ for bone fracture healing [40]; PDA-bredigite demonstrated a strong adhesion ability under a wet condition, and BR NPs accelerated the mineralization of calcined tissues. These hydrogels exhibited strong adhesion (45.9 kPa) to cow skin via reversible non-covalent and irreversible covalent crosslinking and antibacterial properties *ex vivo.* Characteristics of DA-modified GE-based TAs are presented in Table 2.

**Table 2:** The characteristics of DA-modified GE-based TAs

Applications	Components	*In vivo* model	Results	References
Hemostasis	DA, GE, PBA, GO	Rat	Fast hemostatic property with movement monitoring of finger	[29]
	DA, GE, PBA, HA	Rat	Rapid hemostasis with high tissue adhesion	[30]
Wound dressing	GEMA, PDA	Porcine	5.7 times stretchability and 4 times adhesion force compared to those of GEMA	[31]
	GEMA, CO NPs, AMP	Rat	Rapid healing speed and promotion of rat skin restoration	[32]
	PDA, GE, nano-clay	SD rat	Enhanced healing of oral ulcers due to the strong mucoadhesive property	[33]
	DA, GEMA	Mouse	Accelerating wound healing with stretchable and breathable capacities	[34]
	GEMA, QCS, glycerin	Rat	Better hemostasis than a commercial GE sponge	[35]
	EV, DA, GEMA	Rat	Prominent wound healing with expression of IL-6 and collagen deposition	[36]
	GE, silica, DA	SD rat	Significant increase in wound healing rates	[37]
Bone defect repair	BMSC, DA, GE, GEMA, methacrylated SF, GO	SD rat	Enhanced osteogenesis by 3D bio-printed artificial periosteum	[38]
	DA, GE, PDA, HY	Rat	Acceleration of bone repair efficiency	[39]
	P(DO-co-AC), BR, FE^3+^	Cow skin	Acceleration of mineralization of calcined tissues	[40]

##### Advantages and disadvantages

GE-based TAs offer several advantages such as biocompatibility, biodegradability, anti-inflammatory activity, and induction of cell adhesion. Nevertheless, the molecular weight of GE covers a broad range according to collagen denaturation [74], GE-based TAs are prepared using porcine-derived GE and require heat treatment before being used in surgery [75], and porcine-derived GE is gel-like at room temperature because of its high imino acid content.

#### DA-modified chitosan (CS)-based TAs

##### Characteristics of CS

CS fabricated by the deacetylation of chitin, mainly found in the exoskeletons of animals and cell walls of fungi, has been extensively applied in drug delivery carriers [41], wound dressings [42], and tissue engineering [43, 44] owing to its biodegradability, biocompatibility, antibacterial property, adjustable formulations, and facile chemical modification [45]. Due to these properties, CS has attracted attention for application in TAs.

##### Applications

*Hemostasis*

Yang et al. prepared multifunctional TA CS hydrogels comprising methacrylate anhydride DA (DAMA), Zn-doped whitlockite NPs, and MA quantized CS (QCSMA) via photopolymerization and coordination of Zn with DA for liver hemostasis and infected wound healing [46]; double bond covalent crosslinking of QCSMA and MADA acted as the first crosslinking network of the hydrogels, and coordination of Zn ions with DA group, stacking of DAMA benzene rings, and H bonding served as the second non-covalent crosslinking network. These hydrogels demonstrated appropriate adhesions (31.0 kPa) with hemostasis, disinfection, and low hemolysis ratios *in vitro*. Furthermore, they exhibited excellent hemostatic effects (129 ± 22 s) in the hemorrhaging liver of SD rat and the fastest wound closure ratio throughout the treatment period in a rat model of full-thickness skin defect owing to the inherent antibacterial property of QCS and Zn ions released as coagulation factors from Zn-doped whitlockite [47].

*Wound dressing*

Zhang et al. prepared flexible bilayer TA hydrogels comprising poly (vinyl alcohol) (PVA), DA-CS, and poly(acryl amide) (PAAm) for strain sensors [48]; PVA as the upper hydrogel layer effectively enhanced the mechanical properties of the hydrogels, and DA-CS exhibited adhesive property with outstanding antibacterial effects. PAAm also appropriately adhered to the skin surface. These hydrogels demonstrated excellent adhesions (0.6 kPa) to human skin, tensile strains of 218.0%, antibacterial property, and conductivity of 1.65 S m^−1^ *in vitro*. Furthermore, they enhanced wound healing in a whole skin defect mouse model due to the synergistic influence of DA and CS in inhibiting the inflammatory response and promoting angiogenesis [49], implying significant potentials of these hydrogels for application in wearable electronic wound dressings.

Cai et al. synthesized platelet-derived growth factor (PDGF)-loaded TA hydrogels composed of methacrylamide CS and DA-conjugated four-arm PEG acrylate via photo-cross-linking for wound dressing [50]; PDGF released from the hydrogels efficiently promoted wound healing owing to the stimulation of cell proliferation and migration, and cross-linked CS and DA-conjugated four-arm PEG hydrogels exhibited excellent mechanical, adhesive, and hemostatic properties. These hydrogels demonstrated superior mechanical and hemostatic properties in *in vitro* blood clotting and rat liver hemorrhage assays. Additionally, they exhibited faster wound closure and collagen maturation in full-thickness skin excisions of SD rats, demonstrating considerable potential for wound dressing applications.

Song et al. prepared a multifunctional TA hydrogel comprising DA, MA-QCS, and poly(vinyl pyrrolidone) (PVP) via UV irradiation for wound dressing [51]; QCS not only exhibited hemostatic property and prevented infection, but also created a favorable moist condition; DA contributed to the outstanding adhesive property of the hydrogel, and PVP afforded H bonds and demonstrated hydrophobic interactions with the tissue surface. This hydrogel exhibited antioxidant property with adequate adhesion *in vitro *due to DA. Moreover, it demonstrated fast hemostasis in SD rat tail amputation and liver bleeding models with excellent and reversible adhesive properties (12.23–24.31 kPa), exhibiting potential for application in the rapid and efficient hemostasis of inflammatory wounds.

*Skin regeneration*

Panwar et al. fabricated conductive and injectable TA hydrogels composed of CS, cellulose, and PDA for bioelectronics and tissue regeneration applications [52]; the combination of PDA-conjugated aldehyde cellulose and carboxyl methyl CS (MCS) formed hydrogels with tunable, conductive, injectable, and adhesive properties via ionic, imine, H, and π-π bondings. These hydrogels demonstrated conductivity of 0.01–3.4 × 10^–3^ S cm^−1^ owing to ionic conductivity without a conductive material. Furthermore, complete regeneration of injured rat skin was achieved with extensive collagen deposition when the hydrogels were implanted into SD rats, indicating a promising application of these hydrogels in bioelectronics for tissue regeneration.

*Spinal cord injury treatment*

Liu et al. prepared DA-modified CS TA hydrogels with outstanding cell compatibility and antioxidant properties comprising DA, CS, and citric acid by crosslinking DA-CS with citric acid for spinal cord injury treatment [53]; inferior mechanical properties of DA-CS were enhanced by crosslinking it with citric acid. DA-CS improved cell survival and adhesion *in vitro* as compared to the case of CS. Moreover, after being implanted into the injured spinal cord of rat, the hydrogels modulated immunity and promoted macrophage polarization to the M2 phenotype and axonal regeneration, suggesting a new strategy for treating spinal cord injury.

*Bone regeneration*

Wu et al. synthesized BML-284-loaded sandwich-like hybrid TA scaffolds composed of beta-tricalcium phosphate (β-TCP), PDA, BML-284, and CMCS for bone regeneration [54]; β-TCP provided a biomimetic 3D porous microenvironment, released BML as the Wnt signaling activator facilitated the adhesions, migrations, proliferations, and osteogenic differentiations of MC3T3-E1 cells, PDA promoted high-efficiency drug loading into the scaffolds, and negatively charged CMCS exhibited electrostatic interaction with positively charged drugs [55]. These hybrid scaffolds enhanced the angiogenic activity of human umbilical vein endothelial cells and suppressed osteoclastic activity with osteogenesis and angiogenesis *in vitro*. Additionally, they stimulated the polarization of M2 macrophages and recruited endogenous stem cells at the injury site to accelerate bone ingrowth and angiogenesis in a critical-sized cranial defect rat model, demonstrating strong potentials for clinical application in orthopedic implants.

Characteristics of DA-modified CS-based TAs are presented in Table 3.

**Table 3:** The characteristics of DA-modified CS-based TAs

Applications	Components	*In vivo* model	Results	Ref
Hemostasis	CS, DA, MADA, Zn whitlockite, MAQCS	SD rat	Excellent hemostatic effect of 129 ± 22 s, the fastest wound closure ratio	[46]
Wound dressing	MSC, ulvan, CS, DA, silver	Mouse	Acceleration of skin wound healing of type II diabetic mellitus mouse	[48]
	PDA, DA, CS, PAAm	Mouse	Conductivity of S.M-1 in vitro, inhibition of inflammatory response and promotion of angiogenesis	[50]
	PDGF, methacrylamide CS, DA, PEG acrylate	SD rat	Faster wound closure and collagen maturation	[51]
	DA, MA-QCS, PVP	SD Rat	Fast hemostatic property with reversible adhesion property	[52]
Skin regeneration	CS, cellulose, PDA, MCS	Rat	Conductivity of 0.01–3.4 × 10−3S cm−1, complete regeneration of rat skin with collagen deposition	[53]
Spinal cord injury	DA, CS, citric acid	Rat	Promotion of macrophage polarization to the M2 phenotype and axonal regeneration	[54]
Bone regeneration	BML-284, β-TCP, PDA, CMCS	Rat	Acceleration of bone ingrowth and angiogenesis	[55]

##### Advantages and disadvantages

CS-based TAs exhibit numerous advantages such as biodegradability, biocompatibility, antibacterial activity, applicability in various formulations with sponges, bandages, and hydrogels, and many chemical modifications with hydrophilic/hydrophobic groups. However, the physicochemical and biological properties of CS cannot be precisely controlled because they depend on the biological sources, molecular weight, and degree of acetylation of CS.

#### DA-modified alginate (AL)-based TAs

##### Characteristics of AL

AL obtained from brown seaweeds consisting of α-L-guluronic acid (G-blocks) and β-(1–4)-D-mannuronic acid (M-blocks) has been widely applied in tissue engineering, wound dressing, cell therapy, and drug delivery [56] because it is non-toxic, biostable, and biocompatible with facile formulation into gels, films, gauzes, fibers, and wafers [57].

##### Applications

*Hemostasis*

Zhang et al. prepared nanocomposite TA hydrogels comprising DA, OAL, ε-polylysine (ε-PL), and AM for infected wound repair [58]; DA-grafted OAL improved adhesive and hemostatic properties, ε-PL with antibacterial property formed a nanocomposite with DA-grafted OAL via ionic bonding, and AM cross-linked the nanocomposite via radical polymerization, thereby increasing the mechanical strengths of hydrogels. These hydrogels adhered to the bleeding surface in a rat hemorrhaging liver model, leading to an arrest of bleeding in 30 s. Moreover, they accelerated the healing of infected full-thickness rat wounds when compared with the case of a commercial AL sponge, demonstrating potential as multifunctional dressings for promoting the healing of infected wounds.

Quyang et al. synthesized rapidly degrading TA hydrogels composed of DA-OAL, CMSC, amino-modified montmorillonite (AMM), and PVP for wound hemostasis [59]; DA-OAL provided photothermal and adhesion effects [60], CMCS exhibited outstanding antibacterial property with adequate water solubility, PVP endowed the hydrogels with superior mechanical properties by forming numerous H bonds with other substances, and AMM demonstrated excellent hemostatic property. These hydrogels exhibited outstanding tissue adhesion to pig skin under dynamic stretching, twisting, and bending. Furthermore, they promoted hemostatic property in the femoral artery of a SD rat model when compared with the case of Surgiflo™ as a control, demonstrating promising potentials for emergency hemostasis.

*Wound dressing*

Qiao et al. prepared antibacterial conductive self-healing TA hydrogels comprising DA-OAL, CMCS, Fe^3+^, and poly(thiophene-3-acetic acid) (PTA) for infected wound healing [61]; these hydrogels initially produced a cross-linked network via the formation of Schiff bases between DA-OAL and CMCS, and then, the mechanical properties of the hydrogels were further enhanced by adding Fe^3+^; PTA endowed the hydrogels with conductivity, tissue adhesion, and photothermal properties. These hydrogels exhibited suitable mechanical, antioxidant, tissue adhesive, and hemostatic properties and excellent conductivity *in vitro* after near-infrared (NIR) irradiation. Moreover, the sizes of the infected full-thickness defect skin wounds in mice were substantially smaller than those in the case of the Tegaderm™ film after 14 days of treatment, rendering these hydrogels promising candidates for wound healing dressing.

Chen et al. fabricated injectable universal dual-network TA hydrogels composed of polyethyleneimine (PEI), poly(acrylic acid) (PAA), DA, and AL for bioadhesive wound dressing [62]; PEI-PAA complexes offered mechanically reinforced cohesion strengths to these hydrogels, and dual-network hydrogels of PEI-PAA with DA-AL/Ca^2+^ coordination demonstrated synergistic effects of mechanical properties and better instant adhesion to wet surfaces. These hydrogels exhibited superior adhesions, high injectability, stability, and biocompatibility. Additionally, they promoted closure, epidermal regeneration, and tissue functionalization in a full-thickness rat wound-healing model, demonstrating potentials for application in wound dressing.

Ouyang et al. prepared a multifunctional TA hydrogel patch comprising DA-OAL, CMCS, and PVP for wound healing [63], grafting of DA with OAL enhanced tissue adhesion and facilitated excellent photothermal conversion to promote local temperature elevation, and the resulting patch exhibited antioxidant activity; CMCS provided superior antibacterial ability to this patch, and PVP enhanced the mechanical properties of the patch by developing numerous H bonds. The proposed patch demonstrated outstanding tissue adhesion ability, significant scavenging ability against N- and O-free radicals, and antibacterial activity under NIR laser irradiation. Moreover, it enhanced wound healing rates of the infected full-thickness skin defects in mice, indicating its potential for application in wound healing.

Guo et al. synthesized periodontium-mimicking multifunctional TA hydrogels composed of QMACS and DA-OAL for socket healing [64]; QMACS and DA-OAL were cross-linked by dual-cross-linking via blue light irradiation to achieve a tight connection similar to that of gingiva with tissue adhesiveness, antibacterial property, and angiogenesis ability. These hydrogels exhibited superior hemostatic ability and inhibited oral pathogenic microorganisms *in vitro*. Additionally, they demonstrated superior performances in promoting socket healing in a tooth extraction SD rat model when compared with the case of the control group, exhibiting considerable potentials as clinical tooth extraction wound dressings.

*Cartilage regeneration*

Zhang et al. prepared cross-linked exosome-loaded injectable TA hydrogels comprising DA-AL, chondroitin sulfate (CDS), regenerated SF (RSF), and exosome for cartilage regeneration [65]; DA-AL demonstrated high adhesion under wet conditions, CDS relieved pain and promoted cartilage regeneration [66], RSF enhanced the adhesion due to the presence of lysine and tyrosine in it, and exosomes recruited BMSC migration and inflation and facilitated BMSC proliferation and differentiation. These hydrogels exhibited high adhesion strengths of 121.7 ± 12.3 kPa in a 50% humidity environment at 26 °C, which are comparable to that of clinically used enbucrilate TA. Furthermore, they accelerated cartilage defect regeneration *in situ* and extracellular matrix (ECM) remodeling after being injected into rat patellar grooves, offering a promising approach for cartilage defect regeneration.

*Lung cancer surgery*

Ji et al. fabricated injectable TA hydrogels composed of DA-AL, catalase, and NaIO_4_ for preoperative localization of lung nodules [67]; catalase reduced cell damage caused by hydrogen peroxide produced from the autooxidation of DA [68], and NaIO_4_ as an oxidant provided rapid gelation ability with high tissue adhesion to the hydrogels. These hydrogels demonstrated quick gelation for less than 5 min and enough tissue adhesion between two porcine lung tissue sheets *ex vivo*. Moreover, they increased the bursting pressure of rat lung tissue up to 266 ± 15–385 ± 13 mm-H_2_O, preventing hydrogel rupture and migration during localizing lung surgery, indicating significant potentials of these hydrogels for application in lung cancer surgery.

*Health monitoring*

Jin et al. prepared self-adhesive, ultra-stretchable, self-healing, and strain-sensitive TA hydrogels comprising GO, DA, AL, Fe^3+^, and poly(acrylic acid-co-acrylamide) [P(AA-co-AM)] for strain sensors [69] via one-pot *in situ* copolymerization; GO/DA acted as nanofillers and led to superior adhesiveness and a double networked AL/P(AA-co-AM) matrix cross-linked by radical copolymerization using a crosslinking agent; Fe^3+^ as an ionic bonding agent afforded strong mechanical properties, and chelation between PAA and Fe^3+^ generated self-healing hydrogels with stretchability and flexibility [70]. After optimization of the DA and AL contents, these hydrogels exhibited ultra-stretchability of 1198%, high adhesion strengths of 36.9 kPa, and conductivity of 3.24 S m^−1^. Additionally, they demonstrated no physiological damage, such as erythema, edema, and ulcers, on the skin of the participant arms after 12 h of allergy patch tests, implying that these hydrogels are ideal candidates for strain sensors.

Zhang et al. synthesized stretchable and self-healing TA hydrogels composed of DA-AL, 3-aminophenyl boronic acid (PBA)-grafted AL (PBA-AL), and C nanotubes (CNTs) for expansion–contraction motion monitoring [71]; the covalent bonds formed between PBA and DA endowed the hydrogels with rapid sealing-healing properties, and CNTs afforded substantially high mechanical properties and electrical conductivity to the hydrogels. These hydrogels exhibited high stretchability (500%) and self-adhesiveness to various substrates. Moreover, they monitored subtle expansion–contraction during human breathing and repeatable electrical signals, demonstrating potential applications in health monitoring.

*Pleural or tracheal sealants*

Gasek et al. prepared elastic and durable TA hydrogels comprising DA and AL-MA for pleural or tracheal sealants [72]; DA facilitated effective adhesion of the hydrogels to wet surfaces, and AL-MA provided favorable viscoelastic and mechanical properties to the *in situ* hydrogels easily formed at the wound site by photopolymerization. These hydrogels exhibited excellent adhesion, high mechanical properties, elasticity, and burst pressures without systemic toxicity *in vitro*. Additionally, no air leak was noticed after sealing the induced injuries in *ex vivo* rat lung and tracheal models. Furthermore, these hydrogels demonstrated no evident air leak for up to one-month *in vivo* rat models of pleural injury and for up to two weeks *in vivo* rat tracheal injury models, indicating potential clinical applications of these hydrogels for pleural and tracheal injury patients. Characteristics of DA-modified AL-based TAs are presented in Table 4.

**Table 4:** The characteristics of DA-modified AL-based TAs

Applications	Components	*In vivo* model	Results	Ref
Hemostasis	DA, OAL, ε-PL, AM	Rat	Acceleration of healing on infected full thickness of wounds	[58]
	DA, OAL, CMSC, AMM, PVP	SD rat	Better promotion of hemostatic property compared with Surgiflo™	[59]
Wound dressing	DA, OAL, CMCS, FE^3+^, PTA	Mouse	Much smaller wound size than Tegaderm™ after 14 days	[61]
	PEI, PAA, DA, AL, CA^2+^	Rat	Promotion of closure, epidermis regeneration, tissue functionalization	[62]
	DA, OAL, CMCS, PVP	Mouse	Enhanced wound healing rate in the infected full thickness skin effect	[63]
	QMACS, DA, OAL	SD rat	Superior performance in promoting socket healing in a tooth extraction	[64]
Cartilage regeneration	DA, AL, CDS, RSF, exosome	Rat	Acceleration of cartilage defect regeneration and extracellular matrix remodeling	[65]
Lung cancer surgery	DA, AL, catalase, NaIO_4_	Rat	Increased bursting pressure of lung tissue, migration during localizing lung surgery	[67]
Health monitoring	GO, DA, Fe^3+^, P(AA-co-AM)	Human	No physiological damage on the human arms after allergy test for strain sensor	[69]
	DA, AL, PBA, carbone nanotube	Human	Monitoring subtle expansion–contraction in breathing and repeatable electrical signals	[71]
Pleural and tracheal sealant	DA, AL-MA	Rat	No obvious air leak up to one month of pleural injury and 2 weeks tracheal injury	[72]

##### Advantages and disadvantages

Wound dressings are one of the major applications of AL because a dried AL wound dressing rapidly absorbs wound exudate, offering a moist environment to the wound site for promoting wound healing [73]. Oxidized AL obtained using sodium periodate can be converted to hydrolytically labile AL; nevertheless, AL, as a non-degradable polymer in the body, creates aldehydes that can be used with a crosslinker containing amines for the production of AL-based TAs [74]. However, the G and M block contents in AL depend on the type of natural source, which affects the physicochemical properties of AL. Moreover, ionically cross-linked AL with divalent ions can easily dissolve in water owing to the loss of Ca^2+^ and Mg^2+^.

#### DA-modified hyaluronic acid (HA)-based TAs

##### Characteristics of HA

HA is a non-sulfated anionic linear polysaccharide consisting of alternating β-(1,3)-linked-*N*-acetyl-D-glucosamine and β-(1,4)-linked D-glucuronic acid [75]. HA has been applied for visco-supplementation for arthritis, as a wound dressing, and as a surgical aid in ocular surgery because it regulates viscoelasticity of joint synovial and eye vitreous fluids and tissue hydration due to its high water-binding ability [76, 77].

##### Applications

*Hemostasis*

Han et al. synthesized flexible dual-functionalized TA hydrogels composed of maleic anhydride and DA-HA fabricated via photo-cross-linking of DA-conjugated maleic HA for rapid hemostasis [78]; maleic groups with high substitution provided strong tissue adhesion and *in situ* gelation property to these hydrogels. These hydrogels induced red blood cell aggregation and platelet adhesion *in vitro*. Furthermore, they exhibited superior hemostatic properties in rat liver injury and rabbit femoral artery puncture models as compared to those of commercial gelatin sponge, revealing that these hydrogels are promising bioadhesives for hemorrhage control.

*Wound healing*

Rong et al. prepared a fetal milieu-mimicking atorvastatin (ATV)- and zinc citrate (ZnC)-loaded TA hydrogel comprising DA-HA, DA-grafted chondroitin sulfate (CDS) (DA-CDS), ATV, and ZnC for wound healing [79]; HA and CDS as the fetal ECM accelerated wound healing with hair follicle regeneration, ATV afforded proangiogenic property with tube formation of endothelial cells [80], and ZnC demonstrated significant bioactivities during the hair follicle cycle [81]. These hydrogels exhibited considerable angiogenesis and hair follicle regeneration efficacy, with high hemostatic property *in vitro*. Additionally, they significantly promoted wound healing, and the closure ratio reached over 94% after 14 days of hydrogel treatment, indicating the potential of these hydrogels to simulate the fetal milieu in clinical wound healing.

Yang et al. fabricated injectable, self-healing, and antioxidant TA hydrogels composed of gallic aid (GA)-grafted DA-HA, collagen (CO), and 3-aminophenyl boric acid (APBA)-conjugated γ-poly(glutamic acid) (γ-PGA) for wound repair [82]; CO as the most abundant ECM led to tissue development during wound healing [83], HA induced cellular responses for tissue regeneration, poly-phenols, such as GA-grafted DA-HA, demonstrated antioxidant property owing to their free radical-scavenging capacity [84], and APBA-conjugated γ-PGA offered ROS-scavenging ability [85]. These hydrogels promote cell proliferation and migration with antioxidant and intracellular free radical-scavenging abilities *in vitro*. Furthermore, they facilitated angiogenesis, inhibited inflammation, and enhanced wound healing via CO fiber deposition in a full-thickness skin SD rat model, exhibiting potential for application in wound repair.

Li et al. prepared composite-functionalized TA hydrogels based on DA-grafted oxidized HA blended with QACS by enzyme-catalyzed cross-linking and Schiff base reaction for wound healing [86]; DA-grafted HA provided excellent toughness, adhesion, and tensile property to the hydrogels, and QACS enhanced the physical properties and stabilities of the hydrogels via ionic and covalent bonding with DA-OHA. These hydrogels demonstrated antibacterial, antioxidant, self-healing, and high adhesive properties *in vitro*. Moreover, they efficiently closed the wound and accelerated wound healing in full-thickness skin wound mice by regulating angiogenic proteins and promoting CO deposition, indicative of considerable potential of these hydrogels for wound healing.

Wang et al. constructed injectable TA hemostatic hydrogels based on DA-HA and recombinant human collagen (CO) (rhCO) by the oxidation of catechol groups using a H_2_O_2_/horseradish peroxidase catalytic system for diabetic wound healing [87]; DA-HA endowed these hydrogels with excellent tissue adhesion and hemostatic properties, and rhCO promoted wound repair. These hydrogels exhibited antioxidant, antimicrobial, and hemostatic activity *ex vivo*. Additionally, they demonstrated superior re-epithelialization, granulation tissue formation, and tissue remodeling in a diabetic rat model with full-thickness skin defects, revealing significant potentials of these hydrogels for diabetic wound healing.

*Tissue repair*

Hu et al. prepared multifunctional TA hydrogels by reacting DA-OHA with GE via amidation and loading AgNPs into the resulting hydrogels for abdominal wall defect repair [88]; DA-OHA afforded antioxidant and adhesive properties to the hydrogels, GE promoted cell proliferation, and AgNPs enhanced the antibacterial property of the hydrogels. These hydrogels exhibited outstanding physical and antibacterial properties with high adhesion to pig skin. Furthermore, they accelerated healing by reducing wound inflammation, promoting angiogenesis, and producing granulation tissue in a rat full-thickness abdominal wall defect model, indicating their substantial potential for the treatment of full-thickness abdominal wall defects.

Ge et al. synthesized dynamic self-healing TA hydrogels by host–guest interaction between β-cyclodextrin (β-CD)-modified DA-HA and PEG- and amantadine-modified CMCS and loaded dexamethasone, basic fibroblast growth factor (bFGF), and L-alanyl-L-glutamine (ALG) into these hydrogels for the repair of damaged mucosa caused by ulcerative colitis (UC) [89]; dexamethasone incorporated into β-CD cavities was released to induce M1-to-M2 repolarization of macrophages, co-delivered bFGF and ALG from the hydrogels facilitated the regeneration of ulcerative mucosa, and the reversible host–guest interaction enhanced the persistence of hydrogels. These hydrogels demonstrated firm adhesion to the extracted ulcerative colon tissue of mice after *in situ* implantation *ex vivo*. Moreover, they regenerated the ulcerative mucosa and restored its barrier function to ameliorate UC symptoms in a dextran sodium sulfate-induced acute colitis mouse model, offering an integrative approach for UC treatment.

Xue et al. fabricated highly efficacious dual-network nerve TA hydrogels using DA-isothiocyanate (IC)-modified HA (DA-IC-HA) cross-linked via thiourea-quinone couplings to form the first network and physically self-assembled decellularized nerve matrix (DNM) as the second network for peripheral nerve repair [90]. IC exhibited strong covalent crosslinking with the tissue during polymerization, and DNM is a promising natural material with bioactive peptides and growth factors. These hydrogels demonstrated robust adhesion strength and promoted axonal outgrowth *in vitro*. Additionally, they decreased fibrosis and accelerated axon/myelin debris clearance at 10 days post-surgery as compared to the cases of suture and fibrin glue in a rat-based sciatic nerve transection model, affording a promising method for a rapid and highly efficacious nerve transection treatment to facilitate nerve repair. Characteristics of DA-modified HA-based TAs are presented in Table 5.

**Table 5:** The characteristics of DA-modified HA-based TAs

Applications	Components	*In vivo* model	Results	Ref
Hemostasis	Maleic anhydride, HA, DA	Rat, rabbit	Superior hemostatic property in rat liver and rabbit femoral artery	[78]
Wound dressing	ATV, ZnC, DA, HA, CDS	Mouse	Promotion of wound healing and higher closure ratio of wounded area	[79]
	GA, DA, HA, CO, APBA, γ-PGA	SD rat	Promotion of angiogenesis inhibition of inflammation, enhancement of wound healing	[82]
	DA, HA, QACS	Mouse	Effective closure of wound, acceleration of wound healing	[86]
	DA, HA, γhCO	Rat	Superior re-epithelization, granulation, tissue formation, tissue remodeling	[87]
Tissue repair	DA, OHA, GE, AgNPs	Rat	Acceleration of healing process, promotion of angiogenesis, formation of granulation tissue	[88]
	Β-CD, DA, HA, amantatine, CMCS, DEK, bFGF, ALG	Mouse	Regeneration of ulcerative mucosa, restorement of barrier function of mucosa	[89]
	DA, IC-HA, DNM	Rat	Decrease of fibrosis, acceleration of axon/myelin debris clearance	[90]

##### Advantages and disadvantages

HA-based TAs exhibit several advantages, for instance, they serve as natural moisturizers with high water-binding capacity owing to their highly hydrophilic natures, demonstrate biocompatibility due to their existence in mammalian tissues and non-immunogenicity, and participate in cell–matrix interactions [91]. Nevertheless, HA-based TAs exhibit inferior mechanical properties because of their high swelling and rapid degradation. Moreover, the degradation products of HA induce inflammatory responses in dendritic cells and macrophages [92].

#### DA-modified dextran (DE)-based TAs

##### Characteristics of DE

DE mainly consists of α-1,6-glycosides with some α (1,3) branches and has been applied as a volume expander to treat hypovolemia, coagulation factor to decrease blood viscosity, and inhibitor of thrombocyte aggregation. However, its original form does not contain tissue-reactive groups [74].

##### Applications

*Wound dressings*

Yin et al. prepared multifunctional TA hydrogels based on rosmarinic acid (RA)-grafted oxidized DE and RA-grafted GE via Schiff base reaction for wound dressing [93]; RA demonstrates strong tissue adhesion owing to the same catechol groups as those in DO, DE is biocompatible and non-toxic, and GE promotes wound healing. These hydrogels exhibited short gelation times of 61.6 ± 2.8 s, strong adhesive strengths of 27.30 ± 2.02 kPa, superior storage moduli of 1.31 × 10^4^ Pa, and strong antibacterial property. Furthermore, they demonstrated 4.3 times higher wound healing rates in a rat model of full-thickness skin defects on day 14 of treatment than that in the case of the control group, indicating that these hydrogels are promising candidates as wound dressings.

*Tissue repair*

Chen et al. synthesized injectable *in situ* composite TA hydrogels by mixing oxidized dextran (ODE), CS, and DA via Schiff base reaction and further prepared multi-cross-linked (MC) hydrogels by adding cross-linking agents, such as NaIO_4_ and FeCl_3_, to enhance adhesion via the coordination of Fe^3+^ and DA and non-covalent and covalent bonds of DA for tissue repair [94]; only *in situ* mixing of ODE and CS in the presence of DA provided the injectable and rapid curing adhesives, and sol–gel transition of MC hydrogels was easily adjusted by changing the amounts of the cross-linking agents. The MC-FeCl_3_ hydrogel adhesive exhibited 43 times higher adhesion strength (345 kPa) than that of fibrin glue. Additionally, the hydrogel adhesives demonstrated excellent biodegradability and biocompatibility *in vitro*; nevertheless, *in vivo* studies were not performed.

Characteristics of DA-modified DE-based TAs are presented in Table 5.

##### Advantages and disadvantages

DE-based TAs are mostly employed in oxidized forms with aldehyde groups in the backbone generated via the oxidation of DE by sodium periodate. Aldehyde groups in ODE undergo Schiff base reactions with the amine groups of the crosslinkers, leading to *in situ* gelation. A simple DE sponge can be used as a hemostatic TA via imine bond formation between the aldehyde groups in ODE and amine groups in the tissue after freeze-drying. However, the degradation rate of ODE TA is relatively higher due to the hydrolysis of imine bonds. Moreover, a large amount of DE-based TA is needed to realize adequate sealing and adhesive properties [95].

## Temporary stimulus-responsive TAs for underwater adhesion

Permanent TAs always leave uneven layers when forcibly detached, and peeling off of strong TAs can cause pain in patients. Therefore. temporary stimulus-responsive TAs are necessary to switch between bonding and non-bonding states for achieving on-demand detachment [10]. Smart switchable TAs have been obtained by introducing stimuli responsiveness into numerous DA-modified TAs using pH, light, temperature, and electric field. Related studies are discussed in this section.

### pH-responsive TAs

Narkar et al. fabricated reversible TA hydrogels consisting of DA-MAM, AA, and 3-acrylamido PBA via photo-radical polymerization for pH-responsive adhesion [96]; PAA demonstrated strong adhesion to the substrate at pH = 7.5–8.5; nevertheless, the adhesion considerably reduced owing to the formation of a DA-boronate complex. Interfacial binding property of these hydrogels were successfully tuned by reversibly changing pH. However, the DA-boronate complex should be broken at pH = 3 to recover the elevated adhesive property, indicating that these hydrogels are promising smart adhesives for application in marine pH ranges.

Sieste et al. prepared PDA-coated TA nanofibers based on PDA-coated peptide hybrid nanofibers via peptide nanofiber-mediated auto-oxidative polymerization of DA to stimulate neuronal growth and adhesion [97]; PDA coating on the hybrid nanofibers provided a platform to introduce functionalities during a pH-sensitive polymer analogous reaction. Resulting hybrid nanofibers exhibited high neuronal cell adhesion, and the functional properties of these nanofibers could be reversibly tailored due to the pH sensitivity of PDA.

Arias et al. synthesized artificial mussel-glue proteins based on a histidine-rich domain from preCO in byssus threads with DA and cysteine residues via mussel-inspired polymerization of the histidine-rich domain-peptide-based unimer by chemical activation with NaIO_4_ [98]. These artificial proteins demonstrated strong adsorptions on Al_2_O_3_ owing to the production of β-sheets by moderate changes in pH from 4 to 7; this suggests that the gap between mussel-glue-inspired polymers and mussel foot proteins can be bridged by understanding more complex functions of these proteins.

Hu et al. prepared sprayable antibacterial TA hydrogels by combining zwitterionic sulfobetaine MA (SBMA) and poly (SBMA-co-DAMA)-modified AgNPs (PSBDA@AgNPs), which were polymerized after exposure to UV light, for joint wound treatment [99]; poly SBMA formed PSB chains entangled with the PSBDA@Ag hydrogels at the wound site. These hydrogels killed bacteria in an acidic microenvironment at the infected wound sites via the rapid release of AgNPs. Additionally, they exhibited high wound closure rates on the rat neck skin due to reduced inflammation and enhanced angiogenesis, revealing potential applications of these hydrogels for the promotion of skin wound healing.

### Photo-responsive TAs

Li et al. fabricated injectable TA hydrogels based on DA-HA and PDA-coated Ti_3_C_2_ MXene catalytically cross-linked by an oxyhemoglobin/H_2_O_2_ system combined with photothermal stimulation for diabetic wound healing [100]; oxyhemoglobin functioned as not only a horseradish peroxidase-like catalyst to catalyze the formation of hydrogels, but also an O_2_ carrier to release O_2_ upon being activated by heat via NIR irradiation. These hydrogels scavenged excess reactive N species and ROS and demonstrated superior antibacterial ability owing to the presence of PDA coating. Moreover, they significantly promoted human umbilical vein endothelial cell proliferation and facilitated infected diabetic wound healing in a full-thickness cutaneous injury mouse model upon mild NIR stimulation.

Chung et al. prepared photo-responsive TA hydrogels based on dual networks consisting of DA-HA and SF obtained using tyrosinase as a catalyst for delivering *N*-acetyl-L-cysteine (NAC) from nasal cavity to brain tissues via NIR effect on the NAC-loaded hydrogels [101]; PDA provided NIR photothermal response, and NAC opened tight junctions in the RPMI 2650 cell line, a model cell of the nasal mucosa, as indicated by the decreased transepithelial electrical resistances and enhancement of transepithelial electrical resistances by NIR irradiation *in vitro*. Furthermore, an approximately ninefold increase in the quantity of NAC delivered from the nasal cavity to the brain tissue in the span of 2 h via NIR irradiation in rats was observed for the NAC-loaded hydrogel as compared to the case of the NAC solution as a control because of the photothermal response of the hydrogel.

Ding et al. synthesized sprayable multifunctional TA hydrogels by mixing DA-OHA, cyanoacetate-grafted DE containing black P, and histidine as a catalyst for joint skin wound healing [102]. DA-OHA enhanced tissue adhesiveness with antioxidant capacity, and black P led to the crosslinking of the hydrogels due to the photothermally reversible performances of C=C bonds between aldehyde groups and cyanoacetate groups. These hydrogels exhibited excellent photothermal antibacterial performances and on-demand dissolving ability under NIR irradiation. Additionally, they considerably facilitated mouse joint wound healing owing to the loading of vascular endothelial growth factor into them and their dissolving property under NIR irradiation, indicating their significant potential for the treatment of various motion wounds under different conditions.

### Temperature-responsive TAs

Zhao et al. prepared reversible and temperature-responsive TA hydrogels based on AD- and DA-conjugated PEA as the guest and CD-grafted poly (*N*-isopropyl acrylamide) (PNIPAM) as the host via the host–guest molecular interaction to control the highly tunable superior adhesive property of the hydrogels via temperature modulation [103]; interfacial adhesion was deactivated below the lower critical solution temperature (LCST) and was activated above LCST of CD-grafted PNIPAM. Adhesion of TA hydrogels acquired by host–guest molecular interactions to the wet surfaces was substantially higher at 40 °C than that at 25 °C, and these TA hydrogels demonstrated reversible adhesion strength according to the temperature; nevertheless, *in vitro *and *in vivo* studies were not conducted.

Xu et al. synthesized thermoresponsive TA hydrogels as novel intelligent wet adhesive biomaterials based on PNIPAM by radical polymerization in the presence of DA-CS [104]; PNIPAM exhibits thermoresponsive property. These hydrogels demonstrated reversible sol–gel transition behaviors when the temperature was cycled below and above LCST of 35 °C. Moreover, they exhibited controllable attachment/detachment behaviors over pork skin *ex vivo*. Furthermore, the hydrogel-coated syringe needles demonstrated instant hemostasis after being removed from the punctured sites of mouse veins, revealing the applicability of these hydrogels as new promising intelligent TAs for several biomedical applications.

### ROS-responsive TAs

Huang et al. prepared a stimulation-responsive mucoadhesive probiotic that could specifically adhere to the intestinal inflammatory sites based on DA-phenylboric acid-HA for inflammatory bowel disease (IBD) treatment via the consumption of a large amount of ROS [105, 106]; ROS scavenging offered strong mucoadhesion ability and prolonged the retention times of probiotics in the inflammatory site. ROS-responsive mucoadhesive probiotics considerably alleviated colitis symptoms as compared to the cases of probiotics alone in a murine model of acute and chronic colitis due to their remediation effects, affording a promising strategy for targeted treatment of inflammatory diseases.

### Electrical field-responsive TAs

Bhuiyan et al. investigated the effect of direct application of an electrical field on the adhesive property of DA [107]. Adhesive property of DA-containing Ti spheres serving as conductive electrodes for applying electricity to the adhesive decreased with an increase in the applied voltage and current owing to *in situ* electrochemical oxidation of DA; however, irreversible oxidative crosslinking of DA limits its industrial applications.

### pH- and ROS-responsive TAs

Long et al. fabricated injectable dual-responsive TA hydrogels based on DA-HA and PBA-grafted methyl cellulose for chronic wound repair [108]; DA-HA provided outstanding tissue adhesion under wet conditions, and boronate-ester cross-linked hydrogels were likely to decompose into various reactants under acidic pH or high-ROS conditions [109]. These hydrogels exhibited high antibacterial property, excellent antioxidant activity, and high cell proliferation under acidic pH and high-ROS conditions due to the higher release of AgNPs and ROS from the hydrogel. Additionally, they significantly accelerated wound repair in a diabetic bacterial-infected rat model owing to enhanced CO deposition and granulation tissue regeneration, demonstrating substantial potentials for application in chronic wound dressing.

### ROS- and glucose-responsive TAs

Xu et al. prepared platelet-rich plasma (PRP)-loaded multifunctional TA hydrogels based on PRP, DA-AL, and 6-aminobenzo[c][1,2]oxaborol-1(3H)-ol (ABO)-grafted HA (ABO-HA) via ionic, H, and boronate ester bonds for diabetic wound healing [110]; these hydrogels degraded at high rates to release numerous cytokines from blood platelets under ROS and hyperglycemia conditions due to ROS and glucose dual-responsive property of the boronate ester bonds between ABO and cis-diol of AL or DA [111]. These hydrogels exhibited outstanding injectability, moderate tissue adhesions with excellent hemostasis, and antioxidant property, resulting in low-oxidative stress microenvironments. Moreover, the PRR-loaded hydrogels demonstrated faster complete wound healing than those in the cases of other groups in a full-thickness diabetic wound mouse model established on leptin receptor mutant mice owing to rapid anti-inflammatory activation of the M2 phenotype and promotion of migration and proliferation of fibroblasts, offering an efficient method for chronic diabetic wound dressing.

### pH-, glucose-, and photo-responsive TAs

Zhu et al. constructed intelligent TA hydrogels based on metformin (ME)-loaded PDA NPs, DA-GE, Cu-loaded PDA NPs, and PBA-grafted HA (PBA-HA) via dynamic phenyl borate bonding among composite hydrogels for diabetic infected wound healing [112]; release of ME from the hydrogels was pH- and glucose-dependent to treat different wound microenvironments, and Cu endowed the hydrogels with photothermal responsiveness to kill bacteria and protect wound from infection. These hydrogels exhibited outstanding injectability, excellent adhesion, and ROS scavenging activity and inhibited the activation of nuclear factor kappa B pathway *in vitro*. Additionally, they significantly promoted wound healing in diabetic SD rats due to their antibacterial property, inhibition of inflammation, improvement of angiogenesis, and accelerated deposition of ECM, demonstrating considerable application potentials for diabetic wound healing.

## Essential factors affecting the adhesions of DA-modified TAs

Regulating critical factors for an intended application is an ideal strategy for increasing the adhesions of DA-modified TAs under underwater conditions. In this section, we discuss various physical, geometrical, and chemical factors that can be used to control the adhesions of TAs.

### Physical factors

#### Rigidity

Effect of TA hardness on adhesion has not been adequately explored; nevertheless, as rigidity can affect adhesion, it should be examined. In some cases, DA in a DA-modified TA reduced the rigidity of TA, increased its tolerance to deformation, and enhanced its toughness [113] owing to the many reversible non-covalent interactions arising from DA that dissipate energy under applied force; however, TAs with substantially high rigidity do not adapt to skin tissue deformation.

#### Roughness

Ghorbani et al. analyzed the influence of the chemical composition of the substrate on the homogeneous decorations of PVA/polyurethane-polyaniline matrices with PDA, and lower density of PDA was obtained in bare PVA/polyurethane-polyaniline matrices. Nevertheless, the uniform and dense precipitation of PDA reduced the conductivity of the scaffolds 1.2 times when compared with the cases of the samples with low density of PDA; however, homogeneous decorations of conductive matrices with PDA exhibited suitable cell adhesion and spreading [114].

### Geometrical factors

#### Shapes of TAs

Carbone et al. reported that mushroom-shaped pillars adapted to surfaces with variable roughness and significantly enhanced adhesion via the superposition of van der Waals dispersion force and suction effect because of the configurations of bio-inspired surfaces such as gecko foot pad [115].

Rao et al. designed a TA hydrogel surface with hexagonal facets separated by interconnecting grooves to serve as channels for fast water drainage during contact underwater. Dynamic bonds of the hydrogel formed bridges with the substrate, and the discontinuous hexagonal facets also increased the compliance of the hydrogel and prevented continuous crack propagation via the interface [14]. These two effects led to strong yet reversible adhesion by enhancing the bulk gel energy dissipation and delaying interfacial debonding, as in bio-inspired clingfish.

### Chemical factors

Several chemical factors, for example, surface charge, pH sensitivity, hydrophobicity, hydrophilicity ratio, and type of chemical structure, affect the adhesions of DA-modified TAs. This section briefly reviews the possible interactions between DA and various functional groups. Hydroxyl groups of DA develop H bonds with hydrophilic surfaces. Moreover, the benzene ring of DA demonstrates a π-π stack interaction, cation-π interaction with a positive surface, and hydrophobic interaction with a hydrophobic surface. Furthermore, oxidized DA produces coordination complexes with metal oxide surfaces and covalently bonds with nucleophilic surfaces via Michael addition [116].

## Discussion and future directions

Recently, the use of TAs in underwater environments has attracted the attentions of scientists and industrialists because TAs can be employed in specific applications such as wound dressings, antibacterial agents, wearable devices, bioelectronic devices, biosensors, and water pipeline leakage repair. Nevertheless, wet adhesion technologies and diverse and flexible adhesive materials should be considered to achieve wet adhesion because underwater bonding is substantially different from bonding in air as interfacial water molecules considerably weaken the intimate contact adhesion between the adhesive and submerged surfaces, thereby significantly limiting the application of adhesives in numerous fields. Among various TAs, mussel-inspired TAs have been extensively investigated because of their several advantages such as many interactions with substrates, various designs of some interesting smart TAs, and excellent adhesion based on several interfacial interactions dominated by DOPA, a catecholic amino acid in mussel adhesive proteins.

DA-modified PEG-based TAs are used in wound dressings, bone tissue engineering, and electronic devices due to their biocompatibility and easy chemical modification of their functionality. However, they exhibit numerous disadvantages, for instance, weak mechanical properties, low cohesive strengths with brittleness, high swelling property owing to their hydrophilic natures, and multiple preparation steps, hindering their applications. DA-modified GE-based TAs are employed in hemostasis, wound dressing, and bone defect repair because of their biocompatibility, biodegradability, anti-inflammatory activity, and ability to induce cell adhesion. Nevertheless, they demonstrate several drawbacks such as a broad range of molecular weight of GE according to collagen denaturation, and the requirement of heat treatment of GE-based TAs before use in surgery due to the application of porcine-derived GE that turns gel-like at room temperature because of its high amino acid content. DA-modified CS-based TAs are used in hemostasis, wound dressing, skin regeneration, spinal cord injury treatment, and bone regeneration owing to their biodegradability, biocompatibility, antibacterial activity, applicability in various formulations with sponges, bandages, hydrogels, and chemical modifications with hydrophilic/hydrophobic groups. However, they exhibit disadvantages including difficulty in regulating the physicochemical and biological properties of CS due to the dependences of these properties on the biological sources, molecular weight, and degree of acetylation of CS. DA-modified AL-based TAs are utilized in hemostasis, wound dressing, cartilage regeneration, lung cancer surgery, health monitoring, and pleural or tracheal sealants because of the rapid absorption of wound exudate, providing a moist environment to the wound site for promoting wound healing, and facile conversion of oxidized AL into hydrolytically labile AL. Nevertheless, they demonstrate disadvantages such as the contents of the G and M blocks in AL influence the physicochemical property of AL and ionically cross-linked AL with divalent ions easily dissolves in water owing to the loss of Ca^2+^ and Mg^2+^. DA-modified HA-based TAs are used in hemostasis, wound dressing, and tissue repair due to their high water-binding capacity arising from their highly hydrophilic natures, biocompatibility owing to their existence in mammalian tissues, non-immunogenicity, and participation in cell–matrix interactions. However, they exhibit disadvantages including inferior mechanical property due to their high swelling and rapid degradation of HA and induction of inflammatory responses in dendritic cells and macrophages by the degradation products of HA. DA-modified DE-based TAs are used in wound dressing and tissue repair because of their *in-situ* gelation by Schiff base reactions between the aldehyde groups in ODE and amine groups of the crosslinkers and the preparation of a simple sponge as a hemostatic TA via imine bond formation between the aldehyde groups in ODE and amine groups in the tissue after freeze-drying. Nevertheless, they demonstrate disadvantages, for example, high degradation rates owing to the hydrolysis of imine bonds and the requirement of a large amount of DE-based TA to obtain adequate sealing and adhesive properties. Overall, GE-, CS-, AL-, HA-, and DE-based TAs are biocompatible, non-immunogenic, less toxic, and injectable. GE-, CS-, HA-, and DE-based TAs are degradable, whereas AL- and PEG-based TAs are non-degradable. Sources of CS and AL affect the physicochemical and biological properties of CS and AL. CS, AL, HA, and DE endow TAs with antibacterial, wound-healing, tissue adhesive, and hemostatic property, respectively (Table 6).

**Table 6:** The characteristics of DA-modified DE-based TAs

Applications	Components	*In vivo* model	Results	Ref
Wound dressing	RA, ODE, GE	Rat	Strong adhesive strength of 27.30 ± 2.02 kPa, 4.3 times higher wound healing rate than control group	[93]
Tissue repair	ODE, CS, DA, NaIO_4_, FeCl_3_	NT	Injectable and rapid curing adhesives 43 times more adhesion strength than fibrin glue	[94]

Temporary stimulus-responsive TAs are sometimes necessary to switch between bonding and non-bonding states for achieving on-demand detachment because permanent TAs always leave uneven layers when forcibly detached and peeling off of strong TAs can cause pain in patients. Smart switchable TAs have been obtained by introducing stimuli responsiveness into numerous DA-modified TAs using pH, light, temperature, electric field, and a mixture of two or three components.

From a clinical translation perspective, the primary concerns regarding DA-based materials involve their biocompatibility, biodegradability, and the robustness of their adhesive performance. The oxidation of DA into quinone moieties is accompanied by the release of ROS, which can induce significant cellular oxidative stress. To mitigate these effects, several strategies have been implemented, including removing residual free (non-grafted) DA, and incorporating antioxidants to inhibit H_2_O_2_ formation. While PDA is known to undergo degradation via oxidative enzymes, the specific underlying mechanisms and pathways remain underexplored. Furthermore, the high reactivity of DA moieties poses challenges for post-fabrication processes, such as sterilization. Consequently, rigorous purification during fabrication is essential, and DA-modified TAs must be stored under sub-zero conditions to maintain their functional integrity. Regulating the essential factors, such as physical and chemical factors, affecting the DA-modified TAs for an intended application is an ideal strategy to increase the adhesion and biocompatibility of these TAs under underwater conditions.

Currently, a significant gap exists between research on DA-modified TAs and that on clinically approved products because clinically approved DA-modified TAs are not available at present. However, PEG-, CS-, AL-, and HA-based TAs have been approved for clinical use. To overcome this, designing DA-modified TAs with a deep understanding of the physicochemical properties of the biomaterials, tissue target surface properties, adhesion mechanisms, tissue responses, long-term performances, and clinical and economic limitations is necessary. Additionally, close collaboration among materials researchers, molecular scientists, and clinicians is required. Moreover, an understanding of the developmental pathways and regulations in clinical trials should be considered.

## Data Availability

Data will be made available on request.
